# Supplementary figures and images for: Determinants of institutional maternity services utilization in Myanmar
Source: PLoS One. 2022 Apr 25;17(4):e0266185. doi: 10.1371/journal.pone.0266185 (PMC9037929; doi:10.1371/journal.pone.0266185)

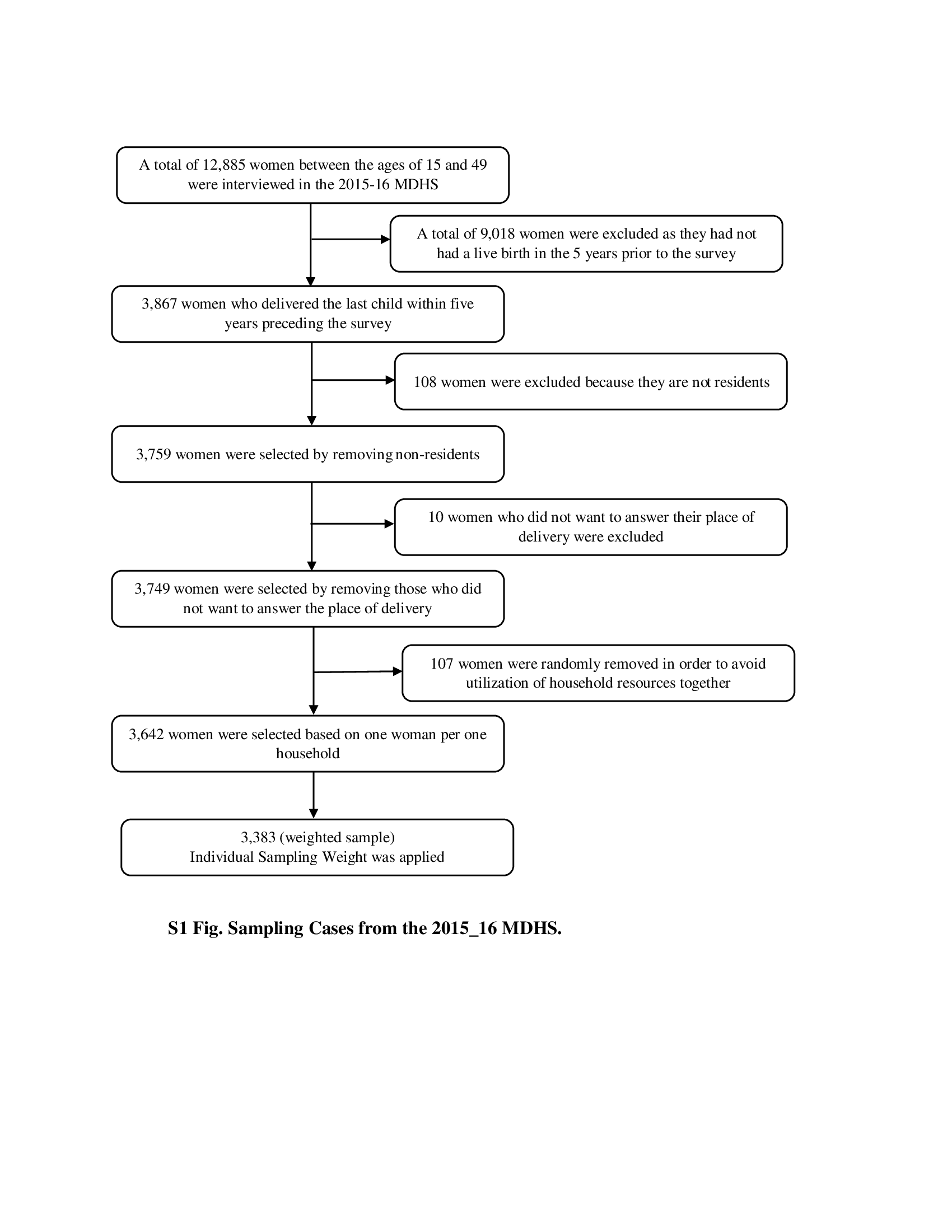

Supplement: S1 Fig — (TIF) [file pone.0266185.s001.tif]
