# Supplementary material for: Determinants of institutional maternity services utilization in Myanmar
Source: PLoS One. 2022 Apr 25;17(4):e0266185. doi: 10.1371/journal.pone.0266185 (PMC9037929; doi:10.1371/journal.pone.0266185)
Supplement: S1 Table — (PDF) [file pone.0266185.s002.pdf]

S1 Table. Operational Definition and Measurement of Variables.

Variables		Measurements
**Outcome variable**		
1	Place of delivery	Institutional delivery (code 1),<br>Non-institutional delivery (code 0)
**Institutional facility availability and accessibility variables**		
2	Urban/Rural	Urban*, Rural
3	States/Regions	Yangon*, Kayah, Kayin, Chin, Sagaing, Tanintharyi, Bago, Magway, Mandalay, Mon, Rakhine, Kachin, Shan, Ayeyarwady, Nay Pyi Taw
4	Experience problems with distance to health facility	No*, Yes
5	Experience problems with getting money needed for advice/treatment	No*, Yes
**Need based characteristics variables**		
6	Number of ANC visits	No ANC visit*, 1-3 times, 4 times or more
7	Experience of pregnancy complication	No*, Yes
**Enabling characteristics variables**		
8	Wife's occupation	Managerial/professional*, agriculture, skilled manual, unskilled manual, not working
9	Husband's occupation	Managerial/professional*, agriculture, skilled manual, unskilled manual
10	Household wealth	Wealthier*, Average, Poorer
**Predisposing characteristics variables**		
11	Age of woman at last delivery	<= 24*, 25-34, 35+
12	Wife's education	No education*, primary, secondary, tertiary
13	Husband's education	No education*, primary, secondary, tertiary

*Indicates a reference category in this study
