# Supplementary material for: Determinants of institutional maternity services utilization in Myanmar
Source: PLoS One. 2022 Apr 25;17(4):e0266185. doi: 10.1371/journal.pone.0266185 (PMC9037929; doi:10.1371/journal.pone.0266185)
Supplement: S2 Table — (PDF) [file pone.0266185.s003.pdf]

S2 Table. Correlation Matrix

	Urban/ Rural	States/ Regions	Experience problems with distance to health facility	Experience problems with getting money needed for advice/ treatment	Number of ANC visit	Experience of pregnancy complication	Wife's occupation	Husband's occupation	Household wealth	Age of woman at last delivery	Wife's education	Husband's education
Urban/Rural	1											
States/Regions	0.093	1										
Experience problems with distance to health facility	0.218	0.053	1									
Experience problems with getting money needed for advice/ treatment	0.134	-0.041	0.484	1								
Number of ANC visit	-0.262	-0.064	-0.234	-0.189	1							
Experience of pregnancy complication	0.010	-0.043	0.072	0.072	-0.024	1						
Wife's occupation	0.016	-0.036	-0.054	0.022	-0.008	-0.029	1					
Husband's occupation	0.081	0.018	-0.003	0.094	-0.040	-0.001	0.315	1				
Household wealth	-0.015	0.105	0.201	0.320	-0.222	0.001	0.090	0.152	1			
Age of woman at last delivery	0.008	-0.070	0.010	0.048	-0.024	0.170	-0.051	-0.031	-0.025	1		
Wife's education	-0.364	-0.088	-0.230	-0.266	0.363	-0.062	-0.114	-0.148	-0.358	-0.135	1	
Husband's education	-0.344	-0.081	-0.182	-0.222	0.341	-0.059	-0.110	-0.189	-0.309	-0.100	0.555	1
