# Supplementary material for: Determinants of institutional maternity services utilization in Myanmar
Source: PLoS One. 2022 Apr 25;17(4):e0266185. doi: 10.1371/journal.pone.0266185 (PMC9037929; doi:10.1371/journal.pone.0266185)
Supplement: S3 Table — (PDF) [file pone.0266185.s004.pdf]

S3 Table. Description of sample characteristics and utilization of institutional delivery *(N=3642 – Unweighted*) *(N=3383 - Weighted)*

**Exposure variables**	**Sample Characteristics**	
	**Number**	**Weighted Number (%)**
**Institutional facility availability and accessibility**		
**Urban/Rural**		
Urban	808	768 (22.7)
Rural	2,834	2,615 (77.3)
**States/Regions**		
Yangon	210	371 (11.0)
Kayah	263	22 (0.7)
Kayin	254	106 (3.1)
Chin	299	41 (1.2)
Sagaing	281	384 (11.4)
Tanintharyi	236	95 (2.8)
Bago	227	302 (8.9)
Magway	227	261 (7.7)
Mandalay	226	362 (10.7)
Mon	191	112 (3.3)
Rakhine	269	228 (6.7)
Kachin	254	120 (3.6)
Shan	253	436 (12.9)
Ayeyarwady	257	466 (13.8)
Nay Pyi Taw	195	78 (2.3)
**Experience problems with distance to health facility**		
No	2,551	2,454 (72.5)
Yes	1,091	930 (27.5)
**Experience problems with getting money needed for advice/ treatment**		
No	2,077	2,069 (61.1)
Yes	1,565	1,315 (38.9)
**Need-based characteristics**		
**Number of ANC visits***		
No ANC visit	489	438 (13.0)
1-3 times	1,036	960 (28.6)
4 times or more	2,084	1,964 (58.4)
**Experience of pregnancy complication**		
No	3130	2987 (88.3)
Yes	512	396 (11.7)

* There is missing data for such variable

S3 Table. (Continued)

Exposure variables	Sample Characteristics	
	Number	Weighted Number (%)
**Enabling Characteristics**		
**Wife's occupation***		
Managerial/professional	172	135 (4.0)
Agriculture	583	495 (14.7)
Skilled manual	746	732 (21.7)
Unskilled manual	825	835 (24.7)
Not working	1308	1178 (34.9)
**Husband's occupation***		
Managerial/professional	267	208 (6.22)
Agriculture	990	887 (26.5)
Skilled manual	965	961 (28.7)
Unskilled manual	1389	1,291 (38.6)
**Household wealth**		
Wealthier	1,098	1,072 (31.7)
Average	722	641 (19.0)
Poorer	1,822	1,670 (49.4)
**Predisposing Characteristics**		
**Age of woman at last delivery**		
<= 24	933	862 (25.5)
25-34	1842	1,764 (52.1)
35+	867	758 (22.4)
**Wife's education**		
No education	592	563 (16.6)
Primary	1,623	1,558 (46.1)
Secondary	1,153	989 (29.2)
Tertiary	274	274 (8.1)
**Husband's education***		
No education	588	552 (16.6)
Primary	1,410	1,365 (41.1)
Secondary	1,354	1,201(36.1)
Tertiary	218	206 (6.2)

* There is missing data for such variable
