# Supplementary material for: Determinants of institutional maternity services utilization in Myanmar
Source: PLoS One. 2022 Apr 25;17(4):e0266185. doi: 10.1371/journal.pone.0266185 (PMC9037929; doi:10.1371/journal.pone.0266185)
Supplement: S4 Table — (PDF) [file pone.0266185.s005.pdf]

S4 Table. Bivariate Analysis Result ***(N=3383)***

**Exposure variables**	**Place of delivery**		**p-value**
	**Non-Institution (Percent)**	**institution (Percent)**	
**Institutional facility availability and accessibility**			
**Urban/Rural**			
Urban	213 (27.7)	556 (72.3)	<0.001
Rural	1,827 (69.9)	788 (30.1)	
**States/Regions**			
Yangon	123 (33.1)	248 (66.9)	<0.001
Kayah	15 (68.1)	7 (31.9)	
Kayin	61(57.7)	45 (42.3)	
Chin	33 (82.1)	7 (17.9)	
Sagaing	249 (64.7)	136 (35.3)	
Tanintharyi	54 (56.9)	41(43.1)	
Bago	179 (59.3)	123 (40.7)	
Magway	158 (60.6)	103 (39.4)	
Mandalay	193 (53.2)	170 (46.8)	
Mon	68 (61.2)	43 (38.8)	
Rakhine	176 (77.2)	52 (22.9)	
Kachin	80 (66.9)	40 (33.2)	
Shan	297 (68.1)	139 (31.9)	
Ayeyarwady	306 (65.7)	160 (34.3)	
Nay Pyi Taw	47 (60.8)	30 (39.2)	
**Experience problems with distance to health facility**			
No	1,334 (54.4)	1,120 (45.6)	<0.001
Yes	706 (75.9)	224 (24.1)	
**Experience problems with getting money needed for advice/ treatment**			
No	1,089 (52.7)	980 (47.4)	<0.001
Yes	951 (72.3)	364 (27.7)	
**Need-based Characteristics**			
**Number of ANC visits***			
No ANC visit	410 (93.7)	27 (6.3)	<0.001
1-3 times	710 (74.0)	250 (26.0)	
4 times or more	907 (46.2)	1,057 (53.8)	
**Experience of pregnancy complication**			
No	1801 (60.3)	1186 (39.7)	0.9888
Yes	239 (60.3)	157 (39.7)	

* There is missing data for such variable

S4 Table. (Continued)

Exposure variables	Place of delivery		p-value
	Non-Institution (Percent)	institution (Percent)	
**Enabling Characteristics**			
**Wife's occupation***			<0.001
Managerial/professional	52 (38.7)	82 (61.3)	
Agriculture	378 (76.4)	117 (23.6)	
Skilled manual	347 (47.5)	385 (52.6)	
Unskilled manual	593 (71.1)	241 (28.9)	
Not working	667 (56.6)	511 (43.4)	
**Husband's occupation***			<0.001
Managerial/professional	71 (34.0)	138 (66.0)	
Agriculture	637 (71.8)	250 (28.2)	
Skilled manual	410 (42.7)	551 (57.3)	
Unskilled manual	901 (70.0)	388 (30.0)	
**Household wealth**			<0.001
Wealthier	465 (43.4)	607 (56.6)	
Average	370 (57.7)	272 (42.3)	
Poorer	1,205 (72.2)	465 (27.8)	
**Predisposing Characteristics**			
**Age of woman at last delivery**			0.8192
<= 24	562 (61.1)	336 (38.9)	
25-34	1,053 (59.7)	711 (40.3)	
35+	461 (60.8)	297 (39.2)	
**Wife's education**			<0.001
No education	476 (84.7)	86 (15.3)	
Primary	1,077 (69.1)	481 (30.9)	
Secondary	439 (44.4)	550 (55.6)	
Tertiary	47 (17.3)	226 (82.7)	
**Husband's education***			<0.001
No education	454 (82.2)	98 (17.8)	
Primary	921 (67.5)	444 (32.5)	
Secondary	597 (49.7)	605 (50.3)	
Tertiary	27 (13.2)	179 (86.9)	

* There is missing data for such variable
