# Supplementary material for: Determinants of institutional maternity services utilization in Myanmar
Source: PLoS One. 2022 Apr 25;17(4):e0266185. doi: 10.1371/journal.pone.0266185 (PMC9037929; doi:10.1371/journal.pone.0266185)
Supplement: Attachment — Submitted filename: Comments_PLOS ONE.docx [file pone.0266185.s007.docx]

Title: Determinants of institutional maternity services utilization in Myanmar

- Page 16/28: line 295: “multivariate analysis” … bivariate analysis
- Abbreviations list: Please include abbreviation list
